# Diabetes mellitus degenerates cisplatin-induced nephrotoxicity in short hydration method: a propensity score-matching analysis

**DOI:** 10.1038/s41598-022-26454-x

**Published:** 2022-12-17

**Authors:** Yoshitaka Saito, Tatsuhiko Sakamoto, Yoh Takekuma, Masaki Kobayashi, Keisuke Okamoto, Naofumi Shinagawa, Yasushi Shimizu, Ichiro Kinoshita, Mitsuru Sugawara

**Affiliations:** 1 Department of Pharmacy, Hokkaido University Hospital, Kita 14-Jo, Nishi 5-Chome, Kita-Ku, Sapporo, 060-8648 Japan; 2 Laboratory of Clinical Pharmaceutics & Therapeutics, Faculty of Pharmaceutical Sciences, Hokkaido University, Kita 12-Jo, Nishi 6-Chome, Kita-Ku, Sapporo, 060-0812 Japan; 3 Department of Respiratory Medicine, Faculty of Medicine, Hokkaido University, Kita 15-Jo, Nishi 7-Chome, Kita-Ku, Sapporo, 060-8638 Japan; 4 Department of Medical Oncology, Faculty of Medicine and Graduate School of Medicine, Hokkaido University, Kita 15-Jo, Nishi 7-Chome, Kita-Ku, Sapporo, 060-8638 Japan; 5 Laboratory of Pharmacokinetics, Faculty of Pharmaceutical Sciences, Hokkaido University, Kita 12-Jo, Nishi 6-Chome, Kita-Ku, Sapporo, 060-0812 Japan

**Keywords:** Oncology, Cancer

## Abstract

Cisplatin (CDDP)-induced nephrotoxicity (CIN) is dose-limiting. We revealed that co-administration of non-steroid anti-inflammatory drugs and baseline comorbidity of diabetes mellitus (DM) are associated with CIN development in the short hydration method; however, the results were accessorily obtained without appropriate power calculation. This study aimed to demonstrate the influence of DM complications on CIN incidence in a real-world setting. Lung cancer patients receiving CDDP (≥ 75 mg/m^2^)-containing regimens with a short hydration method (n = 227) were retrospectively evaluated. The patients were divided into control and baseline DM complication groups. The primary endpoint was the evaluation of CIN incidence between the groups. Propensity score-matching was performed to confirm the robustness of the primary analysis results. CIN occurred in 6.8% of control and 27.0% of DM patients, respectively, with a significant difference in all-patient populations (*P* = 0.001). In addition, variation of serum creatinine and creatinine clearance significantly worsened in DM patients. Similar results were obtained in a propensity-matched population. Multivariate logistic regression analysis found that DM complication is a singular risk factor for CIN development (adjusted odds ratio; 4.31, 95% confidence interval; 1.62–11.50, *P* = 0.003). In conclusion, our study revealed that baseline DM complications significantly worsen CIN.

## Introduction

Chemotherapy is the main treatment strategy for advanced lung cancer^1,2^, and managing chemotherapy-induced adverse effects is necessary for safe, effective treatment and patient quality of life.

Cisplatin (CDDP) is a key chemotherapeutic agent in treating lung cancer in combination with radiotherapy and surgery^1,2^. In contrast, it induces nausea, vomiting, hearing impairment, neuropathy, and nephrotoxicity^3^. CDDP-induced nephrotoxicity (CIN) is dose-relatedly reversible and is known to be its dose-limiting toxicity^3^. CIN used to occur in 30–40% of the patients^3^. However, the progress of CIN management, including magnesium supplementation, quality antiemetic therapy, and appropriate diuretics administration, significantly reduced its occurrence to 0–10%^3–11^.

Many reports evaluate CIN risk factors, although most were conducted using old CDDP administration methods without sufficient CIN prophylaxis described above, suggesting some do not reflect real-world settings^12–22^. We have revealed that co-administration of non-steroid anti-inflammatory drugs (NSAIDs) and baseline comorbidity of diabetes mellitus (DM) are significantly associated with CIN incidence in the short hydration method, the most advanced CDDP administration method^4^. However, the results were obtained without sufficient power calculation. The prevalence rate of DM is increasing worldwide, and people with prediabetes are also growing^23^. Therefore, the CDDP administration to DM patients is increasing. It is important to manage CIN in higher-risk patients although there is a lack of evidence in the population. This study aimed to determine the real-world impact of DM complications on CIN incidence.

## Results

### Patient characteristics

**Figure 1:**
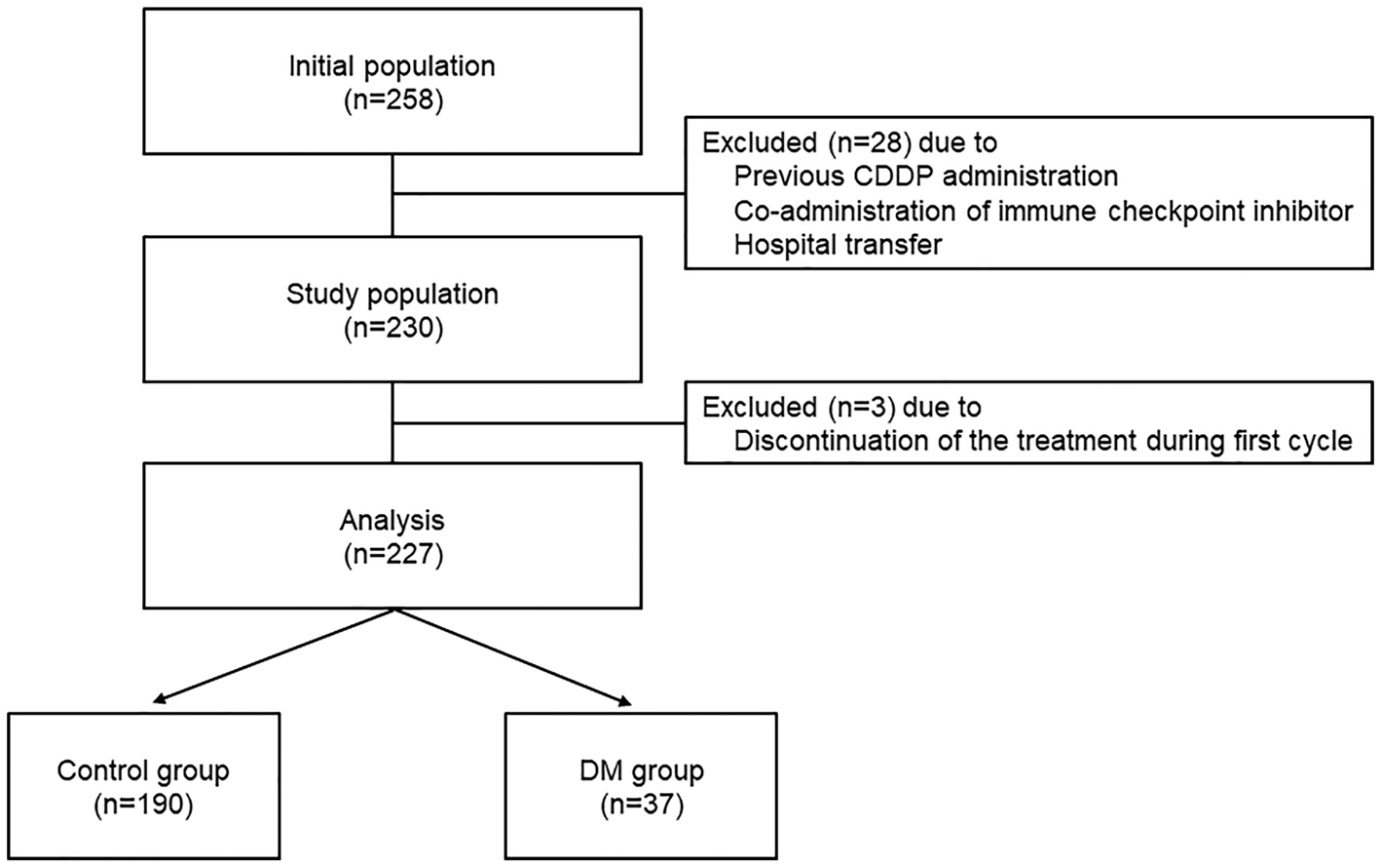
Design of this study.

In total, 227 patients were enrolled in this study based on its eligibility criteria (Fig. 1). The baseline patient characteristics (all-patient population and propensity score-matched population) are shown in Table 1. Patients in the DM group were significantly older and staged earlier than controlled patients. In addition, patients with cardiovascular diseases (controlled hypertension and ischemic heart disease, according to a previous report^19^) were more included in the DM group than control patients. In contrast, no background differences were confirmed between the groups in the propensity score-matched population. Patients with co-administered NSAIDs, an independent CIN risk factor, accounted for 13.7% of the control group and 13.5% of the DM group in all populations, and 6.1% and 12.1% in propensity-matched populations, respectively, without significant difference. Total administration cycles were not different between the groups (median cycles 4 [interquartile range 2–4 cycles] for both groups in the all-patient population, *P* = 0.54; 4 cycles [2–4 cycles] for propensity-matched population, *P* = 0.58). All DM patients were type 2 with well-controlled HbA1c (median 6.6%, range 5.4–9.3%). During the evaluation periods, none of the patients had type 1 DM. Detailed DM medication is shown in Supplemental Table 1.

**Table 1:** Patient characteristics for all patients and propensity-matched patients.

	All-patient analysis			Propensity-matched analysis		
	Control group	DM group	*P*-value	Control group	DM group	*P*-value
No. of patients	190	37		33	33	
Gender: male/female	125/65	28/9	0.34	26/7	24/9	0.77
Median age (range)	63 (32–75)	66 (46–75)	0.04*	64 (38–75)	66 (46–75)	0.70
**Performance status**						
0–1/2	183/7	35/2	0.64	33/0	32/1	1.00
BSA (m^2^) (median, range)	1.66 (1.28–2.00)	1.73 (1.36–2.02)	0.12	1.68 (1.41–1.97)	1.72 (1.36–2.02)	0.69
**Staging**						
Stage IV, Recurrence/Others	85/105	10/27	0.048*	12/21	10/23	0.79
Adjuvant chemotherapy (n, %)	46 (24.2)	9 (24.3)	1.00	8 (24.2)	9 (27.3)	1.00
**Treatment line**						
First line/Second or later line	26/164	4/33	0.79	3/30	4/29	1.00
Albumin (g/dL) (median, range)	4.0 (1.4–4.9)	4.0 (2.3–4.8)	0.43	4.1 (2.6–4.6)	4.0 (2.3–4.8)	0.89
Hemoglobin (g/dL) (median, range)	12.9 (7.7–16.7)	13.0 (10.3–15.6)	0.34	13.0 (10.5–16.0)	13.2 (10.3–15.6)	0.97
Creatinine (mg/dL) (median, range)	0.68 (0.35–1.08)	0.69 (0.40–1.03)	0.42	0.72 (0.40–1.08)	0.69 (0.40–1.03)	0.91
CCr (mL/min) (median, range)	95.8 (51.5–165.1)	95.7 (61.3–136.9)	0.96	89.6 (51.5–155.0)	95.7 (61.3–136.9)	0.51
eGFR (mL/min/1.73 m^2^) (median, range)	78.4 (49.9–140.5)	80.4 (53.5–120.2)	0.93	79.7 (49.9–119.8)	80.4 (53.5–120.2)	0.91
Sodium (mEq/L) (median, range)	140 (125–144)	139 (133–144)	0.36	139 (125–144)	140 (133–144)	0.78
Potassium (mEq/L) (median, range)	4.2 (3.3–5.0)	4.2 (3.1–5.1)	1.00	4.3 (3.8–4.9)	4.2 (3.1–5.1)	0.25
Chloride (mEq/L) (median, range)	104 (92–109)	104 (94–109)	0.34	104 (92–109)	104 (94–109)	0.61
Co-administration of NSAIDs (n, %)	26 (13.7)	5 (13.5)	1.00	2 (6.1)	4 (12.1)	0.67
Co-administration of PPI (n, %)	84 (44.2)	18 (48.6)	0.72	18 (54.5)	15 (45.5)	0.62
Cardiovascular diseases (n, %)	48 (25.3)	20 (54.1)	0.001**	16 (48.5)	16 (48.5)	1.00
**Chemotherapy regimen (n, %)**						
CDDP + PEM	51 (26.8)	7 (18.9)		6 (18.2)	7 (21.2)	
CDDP + PEM + BV	30 (15.8)	1 (2.7)		4 (12.1)	1 (3.0)	
CDDP + VNR	74 (39.0)	16 (43.2)		13 (39.4)	12 (36.4)	
CDDP + ETP	35 (18.4)	13 (35.1)	–	10 (30.3)	13 (39.4)	

*P < 0.05; **P < 0.01.

*DM* diabetes mellitus, *BSA* body surface area, *CCr* creatinine clearance, *eGFR* estimated glomerular filtration rate, *NSAIDs* non-steroid anti-inflammatory drugs, *PPI* proton pump inhibitors, *CDDP* cisplatin, *PEM* pemetrexed, *BV* bevacizumab, *VNR* vinorelbine, *ETP* etoposide.

### Comparison of CIN incidence and variation of serum creatinine levels and creatinine clearance

**Figure 2:**
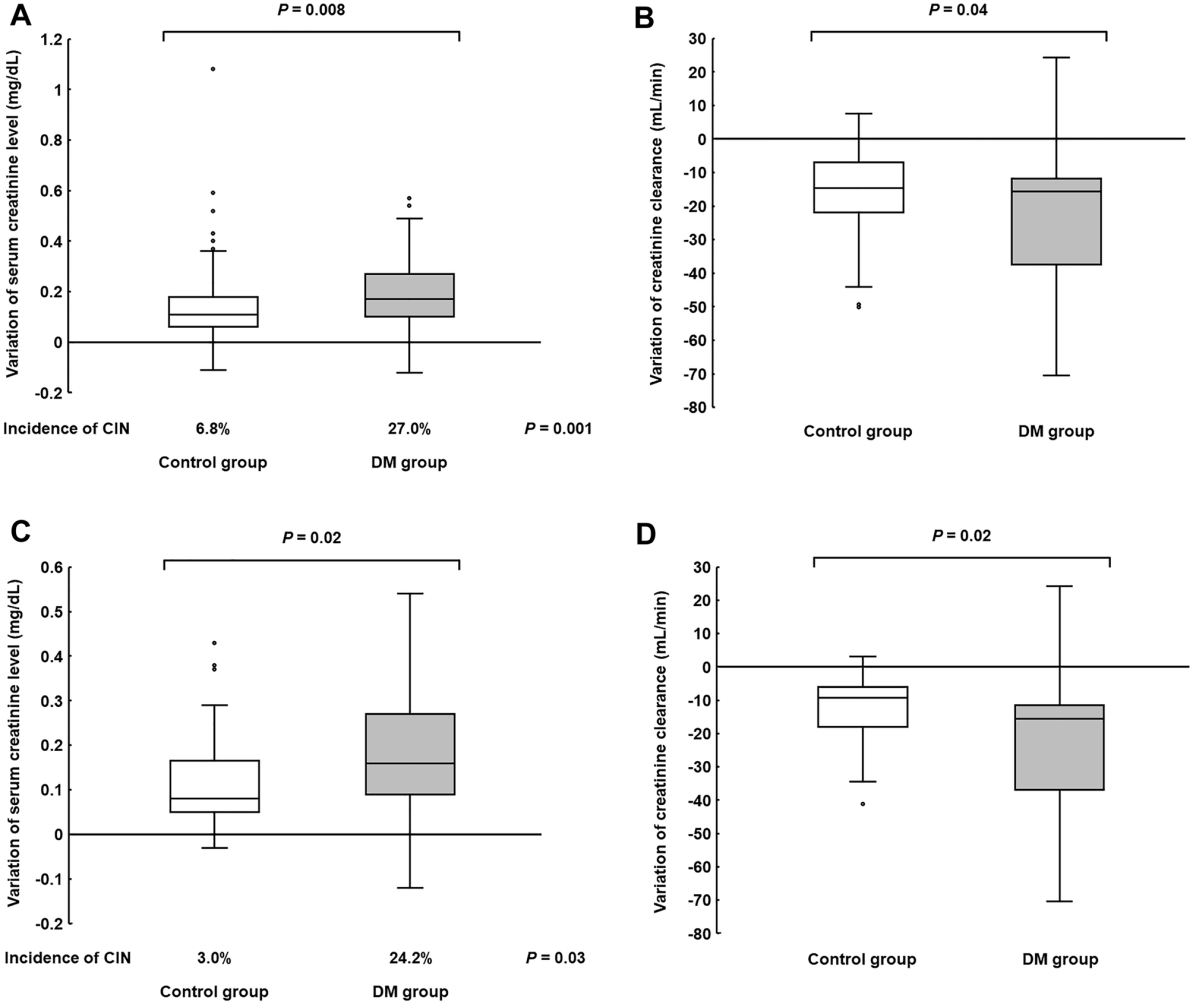
Comparison of (**A**) CIN incidence and SCr variation and (**B**) CCr variation in all-patient population, and (**C**) CIN incidence and SCr variation and (**D**) CCr variation in propensity-matched population. CIN, cisplatin-induced nephrotoxicity.

CIN occurred in 6.8% of control and 27.0% of DM patients, respectively, with a significant difference in the all-patient population, meeting the study's primary endpoint (Fig. 2A). In addition, variation of serum creatinine (SCr) levels and creatinine clearance (CCr) significantly worsened in DM patients (Fig. 2A,B). Similar results were also obtained in propensity-matched populations, with significant differences (Fig. 2C,D).

### Incidence and severity of gastrointestinal symptoms and hearing impairment

Management of chemotherapy-induced nausea and vomiting (CINV) according to current guidelines^24^ is important for sufficient oral hydration, which is an important hydration method. Therefore, in addition to hearing impairment, we also evaluated nausea, vomiting, and anorexia (Table 2). The incidence and severity of gastrointestinal adverse effects and hearing impairment were similar in both populations.

**Table 2:** Incidence and severity of nausea, vomiting, anorexia, and hearing impairment in all subsequent cycles.

	All-patient analysis			Propensity-matched analysis		
	Control group (n = 190)	DM group (n = 37)	*P*-value	Control group (n = 33)	DM group (n = 33)	*P*-value
**Nausea**						
All grade	121 (63.7%)	18 (48.7%)	0.10	17 (51.5%)	16 (48.5%)	1.00
Grade 1	55 (28.9%)	7 (18.9%)		7 (21.2%)	7 (21.2%)	
Grade 2	55 (28.9%)	9 (24.3%)		9 (27.3%)	8 (24.2%)	
Grade 3/4	11 (5.8%)	2 (5.4%)	0.19	1 (3.0%)	1 (3.0%)	0.79
**Vomiting**						
All grade	9 (4.7%)	1 (2.7%)	1.00	0 (0%)	1 (3.0%)	1.00
Grade 1	8 (4.2%)	0 (0%)		0 (0%)	0 (0%)	
Grade 2	1 (0.5%)	1 (2.7%)	0.60	0 (0%)	1 (3.0%)	0.32
**Anorexia**						
All grade	119 (62.6%)	18 (48.7%)	0.14	19 (57.6%)	16 (48.5%)	0.62
Grade 1	53 (27.9%)	6 (16.2%)		7 (21.2%)	6 (18.2%)	
Grade 2	54 (28.4%)	11 (29.7%)		10 (30.3%)	9 (27.3%)	
Grade 3	12 (6.3%)	1 (2.7%)	0.14	2 (6.1%)	1 (3.0%)	0.45
**Hearing impairment**						
All grade	3 (1.6%)	1 (2.7%)	0.51	0 (0%)	1 (3.0%)	1.00
Grade 2	3 (1.6%)	1 (2.7%)	0.51	0 (0%)	1 (3.0%)	1.00

*DM* diabetes mellitus.

### Risk factor analysis for CIN development

Multivariate logistic regression analysis in the all-patient population revealed that DM complication is a singular risk factor associated with CIN development (adjusted odds ratio; 4.31, 95% confidence interval; 1.62–11.50, *P* = 0.003, Table 3). Patients with NSAIDs co-administration had a higher risk of CIN, but this was not statistically significant.

**Table 3:** Univariate and multivariate analyses of the risk factors associated with the incidence of CIN in all-patient population.

	Univariate analysis		Multivariate analysis	
	Odds ratio (95%CI)	*p*-Value	Odds ratio (95%CI)	*p*-Value
**Sex**				
Male/Female	1.12 (0.44–2.85)	0.82	Excluded	–
**Age (years)**				
≥ 65/ < 65	1.36 (0.57–3.24)	0.48	Excluded	–
**Performance status**				
2/0 or 1	2.68 (0.52–13.74)	0.24	Excluded	–
**Staging**				
Stage IV or recurrence/others	0.88 (0.37–2.13)	0.78	Excluded	–
**Adjuvant chemotherapy**				
Present/Absent	1.12 (0.42–2.99)	0.83	Excluded	–
**Radiation combination**				
Present/Absent	1.08 (0.42–2.75)	0.88	Excluded	–
**Prior chemotherapy**				
Present/Absent	0.60 (0.13–2.69)	0.50	Excluded	–
**BSA (m**^**2**^**)**				
≥ 1.5/ < 1.5	2.17 (0.49–9.70)	0.31	Excluded	–
**Hb (g/dl)**				
≥ LLN/ < LLN	0.95 (0.40–2.26)	0.90	Excluded	–
**Alb (g/dl)**				
≥ LLN/ < LLN	0.85 (0.36–2.01)	0.71	Excluded	–
**Co-administration of PPI**				
Present/Absent	0.94 (0.39–2.23)	0.88	Excluded	–
**Co-administration of NSAIDs**				
Present/Absent	2.41 (0.87–6.70)	0.09	2.51 (0.85–7.43)	0.10
**Cardiovascular diseases**				
Present/Absent	2.35 (0.98–5.62)	0.06	1.60 (0.62–4.16)	0.33
**Diabetes mellitus**				
Present/Absent	5.04 (2.01–12.63)	0.0006**	4.31 (1.62–11.50)	0.003**

**P < 0.01.

*BSA* body surface area, *Hb* hemoglobin, *LLN* lower limit of normal, *Alb* albumin, *PPI* proton pump inhibitor, *NSAIDs* non-steroidal anti-inflammatory drugs.

## Discussion

DM is associated with many complications, including renal toxicity, which affects pharmacokinetics and chemotherapy toxicities^25^, and we sometimes administer CDDP to DM patients. Mathe et al. also reported that patients with cardiovascular diseases are at a higher risk for CIN development, and complication of DM and cardiovascular diseases further aggravates CIN^19^. However, they did not evaluate the aggravating action of DM alone, and an advanced CIN management strategy using magnesium supplementation was not conducted in the study. Our previous study suggested baseline DM complication as an independent risk factor for CIN development under CDDP short hydration although there was less power to evaluate the association^4^. This study aimed to evaluate whether CIN more frequently occurs in DM patients.

CIN incidence was significantly higher in DM complicated patients than in non-DM patients. In addition, SCr and CCr aggravation varied significantly in DM patients than in the control patients. These results were confirmed in all and propensity score-matched patient populations. Multivariate logistic regression analysis also suggested that DM complication is a singular risk factor for CIN, although cardiovascular diseases were not associated.

Oral hydration substitutes for intravenous hydration in the short hydration method^4^. Therefore, CINV control is important for sufficient oral hydration. However, the incidence and severity of CINV between the groups suggest that oral hydration may have been similar. Consequently, it was suggested that DM directly worsened CIN.

This is the first report suggesting that DM complication significantly degenerates CIN in advanced management. We should frequently monitor renal function in DM patients and administer additional hydration for early countermeasures in case of CIN incidence.

DM has been reported to accelerate kidney aging^26,27^, and long-term DM degenerates intrarenal arterial sclerosis^28^. Furthermore, CIN worsens in autophagy-deficient mice compared to controls^29^. We showed that celecoxib, a cyclooxygenase-2 selective NSAIDs, exhibits a nephroprotective effect against CIN by activating autophagy and suppressing oxidative stress^30^. These results suggest that autophagy works nephroprotective against CIN. In addition, Sakai et al. have reported that autophagic activity differs in type 1 and 2 diabetic nephropathies, and its induction is significantly suppressed in type 2 DM^31^. Most DM patients are diagnosed with type 2^23^, and all patients in the present study were type 2. Consequently, we speculate that CIN is additively or synergistically worsened by DM-induced renal impairment or autophagy suppression. In contrast, aging was not associated with CIN development in this study. In general, aging reduces renal function^19,26,27^. However, it has been suggested that the organic cation transporter 2 (OCT2), which transports CDDP to the proximal tubule, has a critical role in CDDP accumulation^32,33^, and its expression level reportedly decreases with aging^34^. These suggest that there is compensation between renal function decline and CDDP accumulation reduction due to aging. Further studies evaluating these mechanisms are necessary for better CIN management in DM patients.

This study has some limitations. First, this study was retrospectively conducted with a relatively small population from a single institution. Second, as all DM patients in this study were type 2, and renal pathology differs between type 1 and 2 DM^31^, the results may vary for type 1 DM. Third, in all-patient population analysis, patients in the DM group were significantly older than the control patients. Aging is associated with glomerulosclerosis and arteriosclerosis of intrarenal vessels, causing nephron losss^19^. However, age was not a risk factor, and CIN was more common in DM patients, even in propensity-matched groups in this study. Evaluation of a balanced population in all-patient population analysis was desirable. Finally, it has been suggested that OCT2 has single-nucleotide polymorphisms (SNPs)^35^ and that the 808G > T SNP in OCT2 ameliorates CIN without alteration of disposition^36^. We did not assess patients' genetic backgrounds in this study so they might have affected the results.

In conclusion, our study revealed that DM complications significantly worsen CIN. Therefore, we should monitor DM patients cautiously and evaluate its mechanisms and countermeasures for appropriate CIN management.

## Methods

### Patients

This retrospective observational study enrolled lung cancer patients who received CDDP (≥ 75 mg/m^2^) from May 2014 to December 2021. We evaluated CDDP-including regimens such as CDDP (75 mg/m^2^, day 1) + pemetrexed (PEM, 500 mg/m^2^, day 1) ± bevacizumab (BV, 15 mg/kg, day 1), CDDP (80 mg/m^2^, day 1) + etoposide (ETP, 100 mg/m^2^, days 1–3) ± radiation, and CDDP (80 mg/m^2^, day 1) + vinorelbine (VNR, 20–25 mg/m^2^, days 1, 8) ± radiation.

All patients met the following baseline criteria: (1) age ≥ 20 years; (2) detailed patient information available from medical records; (3) 0 to 2 Eastern Cooperative Oncology Group performance status (ECOG-PS); (4) sufficient renal or liver function for CDDP-containing treatment induction. Patients who were previously administered CDDP, transferred hospital during the chemotherapy, could not complete the first cycle, and those who received dose reduction from treatment initiation were excluded. Patients administered immune checkpoint inhibitors were also excluded as we evaluated direct DM influence on CIN. The patients were divided into two groups: the DM group, which includes patients who required pharmacotherapy for DM treatment at baseline between May 2014 and December 2021, and the controls, who did not exhibit DM during the treatment between May 2014 and March 2021. If the patient had uncontrolled DM, they were treated by diabetologists before treatment induction.

We hypothesized that the CIN incidence would be 10% in the control group and 30% in the DM group, with a patient ratio of 5:1, according to our previous research^4^. To achieve 80% power with an alpha error of 5%, the required sample size was 190 subjects in the control group and 38 subjects in the DM group. Finally, 190 patients in the control group and 37 in the DM group were analyzed for eligibility.

The present study was approved by the Ethical Review Board for Life Science and Medical Research of Hokkaido University Hospital (Approval Number: 022-0107) and was carried out following the Declaration of Helsinki and the STROBE statement. However, given the retrospective nature of the study, informed consent from the subjects was waived by the Ethical Review Board for Life Science and Medical Research of Hokkaido University Hospital.

### Treatment methods of prophylactic supportive care

All patients received the CDDP short hydration method, including 8 mEq of magnesium sulfate administration as described previously^4^. In addition, all regimens included the same antiemetic therapy according to the current guidelines^24^; intravenous palonosetron 0.75 mg on day 1, oral aprepitant 125 mg on day 1 and 80 mg on days 2, 3, and dexamethasone 9.9 mg infusion on day 1 and 8 mg orally on days 2–4.

### Evaluation of CIN and other adverse effects

Toxicities in all subsequent treatment cycles were evaluated under the Common Terminology Criteria for Adverse Events, version 5.0, by physicians and pharmacists. Pharmacists confirmed appropriate CDDP administration, including oral hydration in each treatment cycle. Renal function was assessed based on the SCr variation measured by an enzymatic method. CCr was calculated using the Cockcroft-Gault formula. CIN was defined as grade 2 or higher SCr elevation in this study as well as our previous reports^3,4^. The primary endpoint of this study was the evaluation of CIN incidence between the groups. Secondary endpoints were assessment of SCr and CCr variation and CDDP-related adverse effects. Furthermore, propensity score-matching was performed to adjust the baseline factors between the two groups, and matched data were additionally analyzed to confirm the robustness of the primary analysis results.

### Statistical analysis

The differences in baseline clinical characteristics between control and DM groups were assessed using Fisher’s exact probability test for categorical outcome variables and the Mann–Whitney *U* test for continuous parameters. Incidence of CIN was compared using Fisher's exact probability test, and differences in variation of SCr and CCr between the two groups were assessed using the Mann–Whitney *U* test. Assessment of adverse effects other than CIN was conducted using Fisher's exact probability test for the incidence and the Mann–Whitney *U* test for severity. The univariate and multivariate logistic regression analyses were used to identify the independent risk factor(s) for CIN incidence. We included the following baseline covariates: sex, age, ECOG PS, staging, adjuvant chemotherapy setting, radiation combination setting, prior chemotherapy existence, body surface area (BSA), hemoglobin, serum albumin levels, co-administration of proton pump inhibitors (PPI) and NSAIDs, complication of DM and cardiovascular diseases according to previous reports^4,12–22^. Variables that demonstrated potential associations with CIN incidence in univariate logistic regression analysis (*P* < 0.10) were considered when building the multivariable model. Propensity score-matching was performed using the following baseline variables: sex, age, ECOG PS, staging, adjuvant chemotherapy setting, prior treatment existence, BSA, baseline hemoglobin, albumin, SCr levels, NSAIDs and PPI co-administration, and cardiovascular disease complication. To reduce bias with these potential confounding factors, 1:1 matching (without replacement) in the two groups was achieved using the nearest neighbor method with a 0.20- width caliper of the standard deviation of the logit of propensity scores.

All analyses were performed using JMP version 16.2 statistical software (SAS Institute Japan, Tokyo, Japan). Differences were considered statistically significant when *P*-values were less than 0.05.

### Ethics approval and consent to participate

All procedures performed in this study were carried out in accordance with the ethical standards of the institutional and/or national research committee and the 1964 Helsinki declaration and its later amendments or comparable ethical standards. The study was approved by the Ethical Review Board for Life Science and Medical Research of Hokkaido University Hospital (Approved Number: 022-0107). Given the retrospective nature of the study, informed consent from the subjects was waived by the Ethical Review Board for Life Science and Medical Research of Hokkaido University Hospital.

## Supplementary Information

Supplementary Information.

## Data Availability

The datasets used and/or analyzed in the current study are available from the corresponding author on reasonable request.
