# Supplementary material for: Diabetes mellitus degenerates cisplatin-induced nephrotoxicity in short hydration method: a propensity score-matching analysis
Source: Sci Rep. 2022 Dec 17;12:21819. doi: 10.1038/s41598-022-26454-x (PMC9759552; doi:10.1038/s41598-022-26454-x)
Supplement: Supplementary file 1 — Supplementary Information. [file 41598_2022_26454_MOESM1_ESM.docx]

**Supplemental Table 1. Details of DM medication**

|  | Number of patients (n, %) |
| --- | --- |
| Number of medications  1  2  3  4 or more  Types of medication  Dipeptidyl peptidase-4 inhibitors  Metformin  Insulin  Sulfonylurea agents  　Glinides  Sodium-glucose cotransporter 2 inhibitors  α-glucosidase inhibitors  　Pioglitazone  　Glucagon-like peptide-1 analogs | 16 (43.2%)  11 (29.7%)  8 (21.6%)  2 (5.4%)  31 (83.8%)  15 (40.5%)  9 (24.3%)  8 (21.6%)  3 (8.1%)  3 (8.1%)  1 (2.7%)  0 (0%)  0 (0%) |

The types of medications include duplicates.
